# The efficacy of peer teaching for medical microbiology lectures

**DOI:** 10.15694/mep.2017.000132.2

**Published:** 2017-07-20

**Authors:** Popchai Ngamskulrungroj, Pattarachai Kiratisin, Yodying Dangprapai, Iyarit Thaipisuttikul, Amornrut Leelaporn, Suda Luisirirojanakul, Wannee Kantakamalakul, Navin Horthongkam

**Affiliations:** 1 Department of Microbiology Faculty of Medicine Siriraj Hospital; 2 Department of Physiology Faculty of Medicine Siriraj Hospital

**Keywords:** Microbiology, Communication skills, Undergraduate, Collaborative/peer-to-peer, Lectures/large group

## Abstract

This article was migrated. The article was not marked as recommended.

**Background:** A thorough understanding of infectious diseases is needed by medical professionals; therefore effective microbiological teaching is critical. Although faculty lectures are a convenient means of educating large groups of students, they may fail to engage students and convey an understanding of the subject. Therefore, we developed peer teaching methods based on game-based learning.

**Methods:** A group of student representatives were trained to lecture to a class of 300 third-year medical students via a game show format over a 3-year period (2013-2015).

**Results:** The students reported a higher level of understanding (3.6-4.2 vs 3.6-3.9 out of 5; p < 0.001) and more satisfaction (3.9-4.3 vs 3.6-3.8 out of 5; p < 0.001). Peer teaching also significantly improved the teaching skills of the students (8.9-9.2 vs 8.4-8.7 out of 10; p < 0.001). However, equivalent knowledge outcomes were observed between the two methods and peer teaching demanded more out-of-class time for preparation (44 vs 16 hours for 2013, 49 vs 19 hours for 2014 and 2015).

**Conclusions:** Peer teaching did improve the students’ attitude towards learning and conferred teaching skills, but the learning activity needs adjustment to reduce the out-of-class preparation time.

## Introduction

Understanding the complex interactions between the host and microbes is vital among medical professionals. However, in medical school, most teaching focuses on the host with comparatively little time allocated to microbiology teaching. For example, only 6.5 credits are allocated for microbiology, whereas 71.5 credits are allocated for host subjects (such as anatomy, physiology, and biochemistry) in the preclinical part of our curriculum. Thus, effective microbiology education in the limited time available is critical.

Although small group teaching is ideal, it is not practical when dealing with large groups of students as it would require more staff and space than may be available. By contrast, large group teaching by faculty lectures typically results in limited attention levels and short-term memory of the subject area among the audience, leading to a lower level of understanding of the subject being taught, indicating that it is not an optimal method for skill teaching ( Grauer et al. 2008, Persky and Pollack 2010, Wood 2003).

Peer teaching, also referred to as peer-assisted learning or near-peer teaching, is defined as ‘an educational arrangement in which one student teaches one or more fellow students’ ( Bene and Bergus 2014). Although a recent meta-analysis showed no significance difference in the outcomes between students taught by faculties and those taught by their peers ( Rees et al. 2016), peer teaching does still offer many benefits to students’ learning. For example, peer teaching helps to prepare physicians for their future role as educators, to train leadership skills and confidence, to create a comfortable and safe educational environment, to practice peer feedback as part of their multi-source feedback, and to enhance intrinsic motivation in students ( Bene and Bergus 2014).

Despite the great benefits of peer teaching, it is often practiced unsystematically and informally without staff involvement. This may lead to deviations from standard teaching practices and study outcomes. Moreover, effective-learner students disproportionately gain more knowledge when it is left to chance ( Rees, Quinn, Davies and Fotheringham 2016). Therefore, formalised peer learning under faculties’ supervision can help students to learn more effectively and consistently.

In recent years, gaming approaches have increasingly been used in education ( Drace 2013, Masonjones et al. 2014, Pettit et al. 2015). In general, games are designed for players to achieve feedback using a reward-based approach, to engage via social interactions through an online platform, and to feel less threatening as the games lack any real-life negative impact ( Boeker et al. 2013). Therefore, several studies have attempted to use game-based learning and all showed greatly enhanced engagement of students ( Drace 2013). The benefits of incorporating games into learning activities include improved social skills, a more comfortable learning environment, and enhanced recall of factual knowledge ( Pitt et al. 2015). However, since game-based learning is informal, supplementation with formal learning by faculties is also necessary to ensure that the required level and appropriate type of knowledge is being achieved by students ( Pitt, Borman-Shoap and Eppich 2015).

A variety of peer-teaching methods have been effectively implemented. These methods range from one-to-one teaching to one teacher to a large group of students ( Rees, Quinn, Davies and Fotheringham 2016, Sailer et al. 2010, Secomb 2008). Generally, several core concepts of learning are incorporated into peer-teaching methods. First, activities are generally based on ‘problem-solving’ to motivate students for discussions and eventually develop their ‘critical-thinking’ skills. Second, the topic for discussion must be relevant to their current interests and the activities must be designed to mandate every student to participate to ensure ‘engagement’ of the students. Third, activities are typically run by students sharing ideas with the group; this allows students to learn from the ‘feedback’ of their peers. Finally, these activities are best coordinated under supervision and involve feedback from faculties to ensure ‘learning outcomes’ are met.

Thus, we moved from faculty lectures into peer teaching to improve an efficacy of the learning activities and implemented a game-based approach. We found that lectures given by well-trained peers resulted in a more positive attitude towards microbiology learning among students, with similar knowledge outcomes to those taught by faculties.

## Methods

### Activity design for peer teaching

**Figure 1.**
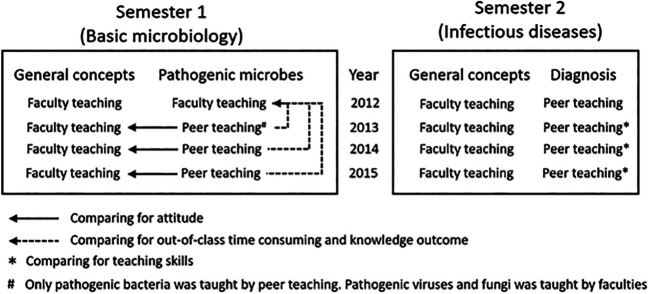
Structure of the microbiology classes and parameters for comparison in this study

Upon approval by our institutional ethics committee (132/2557(Exempt)), we incorporated peer teaching into our microbiology classes replacing selected topics previously taught by faculties. The structure of the microbiology classes is presented in Figure 1. Peer teaching was performed with third year medical students in the Faculty of Medicine at Siriraj Hospital Mahidol University (Bangkok, Thailand) from years 2013 to 2015 (a class of 330 students). The activity design was based on a popular singing contest in Thailand to maximise the attention of students. A representative was selected by their fellow students from each of the 12 small groups in the class. The small groups were arranged independently by our education department. Each representative was tasked with giving a lecture about pathogenic microbes. Different microorganisms were randomly assigned to each representative. The steps involved in the peer-teaching activity are shown in Table 1.

**Table 1.** Correlation between activities and benefits/learning theories

Activities	Benefits	Learning theories
12 medical students volunteered as representatives from each section of a class of 330 students to give lectures in selected topics to the whole class	- To create safe and comfortable education environment as students feels they own the class	- Peer teaching
These volunteered medical students were subjected to be vigorously trained for both microbiological knowledge and presentation skills by faculties.	- To prepare the peer teachers for their future role as educators - To ensure correct content delivery to the class	- Peer teaching
All training sessions were broadcasted and the competitors were promoted by their section peer using a social network. Other students can leave comments for the practice.	- To practice peer feedback as part of multi-source feedback for the peer teachers - To train leadership skills and confidence for the peer teachers	- Peer teaching
Facilitated by faculties, the class teaching was done interactively and mainly by students, At least 2 multiple-choices-question (MCQ) formative evaluations right after each topic using electronic voters.	- To maximize teaching efficacy to class as facilitation was not part of the training - To practice peer feedback as part of multi-source feedback for the peer teachers	- Peer-teaching
The activity was run as a competition which consisted of 3 rounds, qualifying (12-14 students), semi-final (6-7 students) and final rounds (3 students). Selections for winners of each round were done by scoring based on their performance from both medical-educator judges and popular vote from all students. The top three winners and their section peer had their rewards as special scores in microbiology subject and small amount of money.	- To create safe and comfortable education environment as students feels they own the class - To practice peer feedback as part of multi-source feedback for the peer teachers - To enhance engagement of students	- Peer-teaching - Game-based

### Evaluation of the efficacy of peer teaching

1. *Attitude towards peer teaching:* Two parameters were evaluated: understanding the taught-topic and overall impression. These parameters were evaluated by a Likert-scale questionnaire using a rating scale of 1 to 5, where: 1 = very little, 2 = little, 3 = fairly good, 4 = good, 5 = excellent. The question “To what extent did the activity contribute to (parameter of interest)? was posed by the questionnaire. The scores for topics taught by peer teaching were compared with those taught by faculties (see also Figure 1).
2. *Teaching skills:* The teaching skills of the peer teachers were evaluated according to the teaching scores given by the faculties in small-group teaching sessions later in the year. Students were asked to divide into small groups of five students. Each group of students was assigned to give a mini-lecture on infectious disease to a class of 50 students five times. Every student in the group had to participate in the teaching during each session. Teaching scores for each session for each student were evaluated by rating on a 1 (very bad) to 10 (excellent) scale based on the quality of the content and the presentation. The mean scores for the groups with peer teachers were compared with those of the groups without peer teachers (see also Figure 1).
3. *Time commitment:* Out-of-class time, which was spent by faculties for peer teaching and faculty teaching, were compared. The number of hours of preparation and participation in the teaching were calculated (see also Figure 1).
4. *Knowledge outcome:* Knowledge outcomes were determined by the scores from summative evaluations at the end of a semester using multiple choice questions. As the summative scores for the same topics needed to be compared, comparison of the summative scores for the same year with peer teaching was not possible. Splitting of the class into peer-taught and faculty-taught groups was also not possible as our ethics committee advised against such a measure. Therefore, we compared the mean summative scores of the same topics to those of the year 2012, for which the subject ‘pathogenic microbes’ was taught by faculties only. To minimise bias caused by differences in the quality of students each year, the summative test scores were normalised by dividing the scores for the subject ‘pathogenic microbes’ by those for ‘general concepts’ (see also Figure 1).

### Statistical analysis

Statistical analysis was performed using the Mann-Whitney U test or the Student t-test by PSAW program version 18. Statistical significance was designated as p < 0.05.

## Results

### Activities

In the year 2013, a representative for each of the 12 groups within the class of 330 medical students was trained to teach a topic based on pathogenic bacteria for a total of 4 hours (2, 1 and 1 hour for the first, second and third rounds, respectively). In the years 2013 and 2014, topics based on pathogenic fungi and viruses were included in the teaching to a total of 5.25 hours (2, 1.25 and 1 hour for the first, second and third rounds, respectively).

### Students felt that peer teaching gave them a better understanding of the taught subjects and were more satisfied with peer teaching than faculty teaching

Typically, peer teaching creates a positive learning environment that encourages student engagement. We therefore evaluated if such positive feelings were experienced by our students. Using a simple Likert-scale questionnaire, peer teaching was found to result in significantly higher understanding and satisfaction scores, as shown in Table 2.

**Table 2.** Mean scores (out of 5) for each evaluated item on the questionnaire

Year	Type	Understanding the subject	p- value	Overall satisfaction	p-value
2013	Peer-teaching	3.60	0.965	3.89	< 0.001
	Faculty-teaching	3.63		3.62	
2014	Peer-teaching	3.88	< 0.001	4.03	< 0.001
	Faculty-teaching	3.64		3.62	
2015	Peer-teaching	4.24	< 0.001	4.28	< 0.001
	Faculty-teaching	3.85		3.79	

### Peer teachers’ teaching skills were enhanced

As peer teaching is thought to help prepare physicians for their future role as educators, we evaluated if peer teachers developed better teaching skills. We evaluated the teaching skills of the peer teachers by determining their teaching performance at a subsequent peer teaching activity later in the year. The teaching scores of the groups of peer teachers were compared with groups containing no peer teachers. As expected, the groups containing peer teachers had significantly higher teaching scores than groups without peer teachers ( Table 3).

**Table 3.** Comparison of teaching scores

Year	Type	Presentation scores (10)	p- value
2013	Groups with peer-teachers	8.90	< 0.001
	Groups without peer-teachers	8.42	
2014	Groups with peer-teachers	9.24	< 0.001
	Groups without peer-teachers	8.68	
2015	Groups with peer-teachers	9.11	< 0.001
	Groups without peer-teachers	8.67	

### Peer teaching was more time-consuming than faculty teaching

Since peer-teaching activities included training sessions for the peer teachers, the preparation time was expected to be more than that for faculty teaching. Therefore, the out-of-class time taken for teaching preparation was compared, and the results indicated that it took more out-of-class-time for faculties to prepare for peer teaching than that required for faculty lectures (44 versus 16 hours for 2013, 49 versus 19 hours for 2014 and 2015).

### Similar knowledge outcome to faculty teaching was achieved by peer teaching

According to previous meta-analysis, faculty lectures resulted in similar knowledge outcomes to peer teaching. We therefore compared the outcomes of peer teaching to faculty teaching based on the summative evaluation scores. The scores for subjects taught by peer teachers were comparable to those of subjects taught by faculties ( Table 4).

**Table 4.** Comparison of the summative evaluation scores

Year	Mean Score ratio *	Mean Score ratio of 2012 **	p- value
2013	1.22	1.25	0.755
2014	1.11	1.12	0.313
2015	1.17	1.12	0.349

* Scores for topics taught by peer teachers/topics taught by faculty lectures.

** Year without peer teaching, 2012.

## Discussion and conclusion

Peer teaching is being increasingly used in a number of medical schools globally ( Gusic et al. 2013). As the demand for physicians is steadily increasing, the availability of qualified medical educators to teach the next generation of medical students is limited, potentially reducing the quality of student learning. Peer teaching seems to be a valuable tool with which to tackle this problem. However, as the quality of teaching provided by students may be variable, teacher training is mandatory. With proper training, peer teaching has been accepted to be equal to faculty teaching. Therefore, we implemented such measures in the teaching of our microbiology course and the results were promising. The students reported high levels of satisfaction as they felt they could understand the microbiological subjects better after peer teaching than after lectures provided by the faculty. As knowledge outcomes gained by peer teaching was equivalent to those obtained by faculty teaching, peer teaching was considered a promising learning method for our large class size of more than 300 students.

The positive attitude towards peer teaching reported in our study was in line with previous reports ( Mills et al. 2014, Rashid et al. 2011). One previous study reported a positive influence on learning by the implementation of peer learning ( Rashid, Sobowale and Gore 2011). Another study stated a high level of satisfaction towards peer teaching ( Mills, Dalleywater and Tischler 2014). In our study, overall satisfaction was reported to be higher as a result of peer teaching than for faculty teaching. This might be due to the fact that students felt they could understand the microbiological subject better. Such positive feelings towards peer teaching might be due to the more comfortable learning environment that results from having their peers as teachers. The comfortable learning environment created by peer teaching has been well documented in a number of previous studies ( Secomb 2008).

One concern regarding peer teaching lies within the quality of delivery of the content, which directly influences the knowledge outcome of the students. Therefore, we included several training sessions for the representatives targeting both their understanding of the subject matter and their presentation skills. The resulting data for peer teaching showed equivalent knowledge outcomes among the students to faculty teaching. This was consistent with the results of a recent meta-analysis of peer teaching that revealed that peer teaching provided similar knowledge outcomes to faculty teaching ( Rees, Quinn, Davies and Fotheringham 2016).

An additional benefit of peer teaching is that it prepares physicians for their future role as educators ( Kensinger et al. 2015). In Thailand, as in many other countries, medical doctors play an important role as leaders and representatives of the medical profession among communities in rural areas. Typically, Thai medical practitioners are tasked with educating their communities in terms of basic public health knowledge ( Crombie et al. 2005). Therefore, teaching skills are critical for Thai medical doctors but, to date, such training was not officially included in our medical curriculum as a mandatory course. In response, we propose that peer teaching may enhance the teaching skills of our students. We found that peer teachers performed better in teaching sessions later in the year. Moreover, other students in the same teaching groups also displayed improved teaching skills. This suggested that peer teaching did not only increase the teaching skills of the peer teachers themselves but also had a positive effect to those students who interacted with the peer teachers. All peer teachers reported that they trained their fellow students in the teaching skills learnt from our peer teaching activities (data not shown). Taken together, our findings indicate that peer teaching is a promising tool to enhance the teaching skills of future medical doctors in Thailand.

The study provides evidence that peer teaching can be effectively implemented for medical microbiology education. Benefits included high satisfaction levels among students and enhanced teaching skills, without compromising knowledge outcomes. However, a major concern by staff was the fact that the time taken to prepare for such activities was approximately twice as high as that require for faculty teaching. Therefore, adjustments to the activities involved in our peer teaching are warranted before full-scale implementation can take effect.

## Take Home Messages

• Well-organised peer teaching improves attitudes towards medical microbiology teaching
• Training peer teachers for content and presentation skills is crucial for effective large-group lectures as check points for knowledge understanding by reflection and feedback can be limited
• The learning activity for peer teaching has to be designed carefully to ensure that the preparation time is acceptable

## Notes On Contributors

**Popchai Ngamskulrungroj, MD. PhD.**, Assistant professor and a quality manager of microbiology laboratory at Departments of Microbiology, Faculty of Medicine Siriraj Hospital, Mahidol University. Popchai was a committee member of 12 preclinical subjects. He is responsible for medical mycology teaching.

**Pattarachai Kiratisin, MD. PhD.**, Professor and a director of Microbiology Laboratory at Departments of Microbiology, Faculty of Medicine Siriraj Hospital, Mahidol University. Pattarachai was a head of preclinical committee. He is responsible for medical bacteriology teaching.

**Yodying Dangprapai, MD. PhD.**, lecturer at Departments of physiology, Faculty of Medicine Siriraj Hospital, Mahidol University. He is resposible for medical curriculum development of our medical school.

**Iyarit Thaipisuttikul, PhD.**, Assistant professor and a head of postgraduate section at Departments of Microbiology, Faculty of Medicine Siriraj Hospital, Mahidol University. He is responsible for medical bacteriology teaching.

**Amornrut Leelaporn, PharmD. PhD.**, Associate professor and academic manager of microbiology laboratory at Departments of Microbiology, Faculty of Medicine Siriraj Hospital, Mahidol University. She is responsible for medical bacteriology teaching.

**Suda Luisirirojanakul, PhD.**, Associate professor and a safety manager of microbiology laboratory at Departments of Microbiology, Faculty of Medicine Siriraj Hospital, Mahidol University. She is responsible for medical virology teaching.

**Wannee Kantakamalakul, PhD.**, Professor and a deputy director of microbiology laboratory at Departments of Microbiology, Faculty of Medicine Siriraj Hospital, Mahidol University. She is responsible for medical virology teaching.

**Navin Horthongkam, PhD.**, Assistant professor and an academic manager of microbiology laboratory at Departments of Microbiology, Faculty of Medicine Siriraj Hospital, Mahidol University. He is responsible for medical virology teaching.
