# Supplementary material for: The efficacy of peer teaching for medical microbiology lectures
Source: MedEdPublish (2016). 2018 Nov 15;6:132. Originally published 2017 Jul 20. [Version 2] doi: 10.15694/mep.2017.000132.2 (PMC10885281; doi:10.15694/mep.2017.000132.2)
Supplement: Peer review history

# Reviewer report: Reviewer response for version 2

**DOI:** 10.21956/mep.20220.r31313

**Published:** 2019-03-26

**Authors:** Trevor Gibbs

**Affiliations:** 1 AMEE

This review has been migrated. The reviewer awarded 4 stars out of 5 I too would like to congratulate the authors on addressing the comments raised in their previous paper and certainly the paper shows greater clarity now and demonstrates the self-perceived value of peer teaching.One of my other comments from previous was to the degree of value we place upon such a low level of course evaluation. Time does not allow a more longitudinal evaluation in this paper but I hope that the authors can carry on their project and look at how this peer-teaching activity influences later- clinical- learning.

Reviewer Expertise:

NA

No decision status is available

# Reviewer report: Reviewer response for version 2

**DOI:** 10.21956/mep.20220.r31314

**Published:** 2019-03-25

**Authors:** Richard Hays

**Affiliations:** 1 James Cook University

This review has been migrated. The reviewer awarded 4 stars out of 5 I would like to thanks the authors for addressing the comments made by reviewers on the first version of this paper. I think that the comments have been addressed satisfactorily and that this version is improved and worth of citation where relevant. It is noteworthy that per teaching does not appear to be a less expensive method, if done well, but rather may be best regarded as an investment in the development of future teachers.

Reviewer Expertise:

NA

No decision status is available

# Reviewer report: Reviewer response for version 1

**DOI:** 10.21956/mep.19110.r27563

**Published:** 2017-07-31

**Authors:** Julie Browne

**Affiliations:** 1 Cardiff University School of Medicine

This review has been migrated. The reviewer awarded 3 stars out of 5 I enjoyed reading this paper, and congratulate the authors on their work in producing an apparently feasible and pragmatic solution to increasing students’ active participation in learning and teaching in an environment where small group work is a logistical challenge. There were some areas on which I would have liked further information:First, I am not familiar with the game show format and I couldn’t tell from the description how the course was arranged so that all students covered all parts of the syllabus.I would have liked more information about the overall curriculum at this medical school and how this initiative supported the wider educational aims of the institution. How did the student teachers perform in the summative assessments compared with the rest of the cohort? Did the teaching exercise benefit them and if so how?Similar exam scores were achieved by the students regardless of whether they were taught by faculty or peers. Since the peer teaching seemed very labour intensive – it required more than double the time to implement - do the authors consider that this is an intervention that should be pursued, and if so, why? Would the effort taken to train the peer teachers be better applied to developing further the teaching skills of the faculty?Do the authors have any plans to evaluate this study further? In particular, I wonder how the authors will establish that the knowledge that the students gain through the peer teaching will actually translate into good clinical practice. I hope the authors will consider building on this work in a future paper. Thank you for the opportunity to read and comment on it.

Reviewer Expertise:

NA

No decision status is available

# Reviewer report: Reviewer response for version 1

**DOI:** 10.21956/mep.19110.r27560

**Published:** 2017-07-30

**Authors:** Lakshmi Nagappa Naik

**Affiliations:** 1 Melaka Manipal Medical College

This review has been migrated. The reviewer awarded 3 stars out of 5 It was an interesting study especially for PAL in medical education. Regarding a subject like medical microbiology which is exhaustive and has geographical uniqueness with disease spectrum , its clinical significance needs to be emphasised . As this is an active learning method, the close supervision of the contents delivered by peers as teachers is needed. There should be an allocated time for students to be assisted by facilitators prior to presentation and at the end of session a debriefing would help. The article well highlights the elements of success for large group teaching., however the qualitative analysis in form of feedback by all participants will further strengthens the study

Reviewer Expertise:

NA

No decision status is available

# Reviewer report: Reviewer response for version 1

**DOI:** 10.21956/mep.19110.r27561

**Published:** 2017-07-20

**Authors:** James Read

**Affiliations:** 1 Plymouth University Peninsula Schools of Medicine and Dentistry

This review has been migrated. The reviewer awarded 3 stars out of 5 Thank you very much for submitting this paper to the themed edition of MedEdPublish. I read the paper with great interest and I found the format that you had chosen to deliver the peer teaching both innovative and engaging.You describe the development of the project well and the rationale for the use of such an approach is appropriately grounded in recent literature. The scores from students are clearly impressive and it is encouraging to see that they are deriving such a benefit from this approach.There are some areas that I would have liked to have seen more detail on. In particular, how you developed the topics that needed teaching and how these were prioritised over other areas. I also question how much of the benefit is due to the peer teaching and how much is due to an innovative and memorable approach to teaching. I think this significantly confounds your results.I think there are also questions about the impact on students who do not perform as well in their peer feedback and how you have supported these students; both academically and emotionally.I also am always concerned about the use of quantitative statistics when using scales (either 1-10 or likert scales). When using statistical analyses on this data you need to bear in mind that the numbers on the scale mean different things to different people, and that the difference between the numbers may not be constant. Your p values therefore need to be viewed in this context and I would have preferred to see some more qualitative analysis of student comments etc.Overall an interesting paper. Thank you for submitting.

Reviewer Expertise:

NA

No decision status is available

# Reviewer report: Reviewer response for version 1

**DOI:** 10.21956/mep.19110.r27562

**Published:** 2017-07-20

**Authors:** Trevor Gibbs

**Affiliations:** 1 AMEE

This review has been migrated. The reviewer awarded 3 stars out of 5 Any teaching and learning activity that increases active learning seems to derive a benefit for the students and a motivational benefit for the faculty concerned- as such this paper, which increased active learning of both the recipient students and the peer student teachers, was a welcome addition to our increasing knowledge regarding peer-teaching. However it is important to recognise how much importance we place upon the findings from such an activity.-Does the time spent on such an activity outbalance the overall effectiveness of looking at the relevance of microbiology and what emphasis is placed upon the subject. I would have liked to have seen some work initially carried out on what should be taught, rather than as it appears, microbiology is taught as an independent, non integrated block that pays little attention to the relevance and appropriateness of what is taught- is this really active learning or are we just substituting peers for faculty and delivering the programme through the same lecture format?- can we make the positive conclusions given in the paper from the evaluation methodology used, can we move the evaluation further up the line to measure real effect upon learning rather than the low level used in this paperI congratulate the authors in their attempts to improve microbiology teaching but I feel that there is still much to create in this area and would encourage the authors to keep up their enthusiasm in this area

Reviewer Expertise:

NA

No decision status is available
